# *FAAH* rs324420 Polymorphism: Biological Pathways, Impact on Elite Athletic Performance and Insights for Sport Medicine

**DOI:** 10.3390/genes14101946

**Published:** 2023-10-16

**Authors:** Hugo-Henrique Silva, Valéria Tavares, Beatriz Vieira Neto, Fátima Cerqueira, Rui Medeiros, Maria-Raquel G. Silva

**Affiliations:** 1 ICBAS-Institute of Biomedical Sciences, University of Porto, 4050-313 Porto, Portugal; 2 Portuguese Ministry of Education, 1399-025 Lisbon, Portugal; 3 Leixões Sport Clube, Senior Female Volleyball Team, 4450-277 Matosinhos, Portugal; 4 Molecular Oncology and Viral Pathology Group, Research Center of IPO Porto (CI-IPOP)/RISE@CI-IPOP, Portuguese Oncology Institute of Porto (IPO Porto)/Porto Comprehensive Cancer Center (Porto.CCC), 4200-072 Porto, Portugal; i37300@ipoporto.min-saude.pt (B.V.N.); fatimaf@ufp.edu.pt (F.C.); 5 FMUP-Faculty of Medicine, University of Porto, 4200-072 Porto, Portugal; 6 LPCC, Research Department-Portuguese League Against Cancer (LPPC-NRN), 4200-172 Porto, Portugal; 7 FP-I3ID, FP-BHS, CEBIMED and Faculty of Health Sciences, University Fernando Pessoa, 4200-150 Porto, Portugal; raquel@ufp.edu.pt; 8 CIIMAR/CIMAR, Interdisciplinary Centre of Marine and Environmental Research, 4450-208 Matosinhos, Portugal; 9 Pathology and Laboratory Medicine Department, Clinical Pathology SVIPO Porto Portuguese Oncology Institute of Porto, 4200-072 Porto, Portugal; 10 CIAS-Research Centre for Anthropology and Health-Human Biology, Health and Society, University of Coimbra, 3000-456 Coimbra, Portugal; 11 CHRC-Comprehensive Health Research Centre, Nova Medical School, Nova University of Lisbon, 1150-090 Lisboa, Portugal; 12 Scientific Committee of the Gymnastics Federation of Portugal, 1600-159 Lisboa, Portugal

**Keywords:** elite athlete, *FAAH*, gene, polymorphism, sport, performance, success

## Abstract

Gene variation linked to physiological functions is recognised to affect elite athletic performance by modulating training and competition-enabling behaviour. The *fatty acid amide hydrolase* (*FAAH*) has been investigated as a good candidate for drug targeting, and recently, its single-nucleotide polymorphism (SNP) rs324420 was reported to be associated with athletic performance. Given the implications, the biological pathways of this genetic polymorphism linked to elite athletic performance, considering sport type, psychological traits and sports injuries, need to be dissected. Thus, a narrative review of the literature concerning the biological mechanisms of this SNP was undertaken. In addition to its role in athletic performance, *FAAH* rs324420 is also involved in important mechanisms underlying human psychopathologies, including substance abuse and neural dysfunctions. However, cumulative evidence concerning the C385A variant is inconsistent. Therefore, validation studies considering homogeneous sports modalities are required to better define the role of this SNP in elite athletic performance and its impact on stress coping, pain regulation and inflammation control.

## 1. Introduction

An elite athlete can be defined as a highly specialised person in a given sport discipline, possessing exceptional physiological, psychological, physical and environmental (including family, coach, medical and clinical staff) characteristics, allied with an outstanding sports performance [1]. Strong physical and mental preparation with the combination of an adequate training regime, healthy nutrition and close clinical supervision of the athletes’ health are mandatory for elite sport success. In this setting, genetic architecture also plays a major role in athletic performances [2,3,4,5,6,7].

During the past decade, active research on sports genetics has been engaged with various physiological functions linked to cardiovascular, respiratory, nervous and muscle-skeletal systems and their influence on athlete phenotype [1,8]. However, genetic studies on mental abilities affecting resilience, leadership and anxiety and stress management in training and competitions, as well as pain regulation and sports injuries, are scarce [2,3,4,9]. In fact, genes encoding proteins that modulate the operating of the brain’s emotional centre, located in the hypothalamic-pituitary-adrenal (HPA) axis, particularly those related to the production of a stress response, need to be further investigated in the context of sports performance [1]. One of the genes is *fatty acid amide hydrolase* (*FAAH*).

*FAAH* encodes for a key marker of the amygdala-prefrontal cortex circuit that supports emotion regulation. This protein has been mostly studied in rodent models and more recently in humans. Due to its major catabolic activity for the endocannabinoid anandamide (AEA), testing of *FAAH* inhibitors is important for drug development for diverse diseases, including depression, anxiety, aggressive behaviour, borderline personality disorder, substance use disorders and inflammatory bowel disease [10,11,12,13]. Interestingly, in the last five years, the single-nucleotide polymorphism (SNP) *FAAH* rs324420 (also named as c.385C > A or Pro129Thr) has been linked with elite athletic performance in regulating anxiety-like behaviour and influencing persistence and leadership, despite conflicting findings [2,5,6,14].

**Figure 1:**
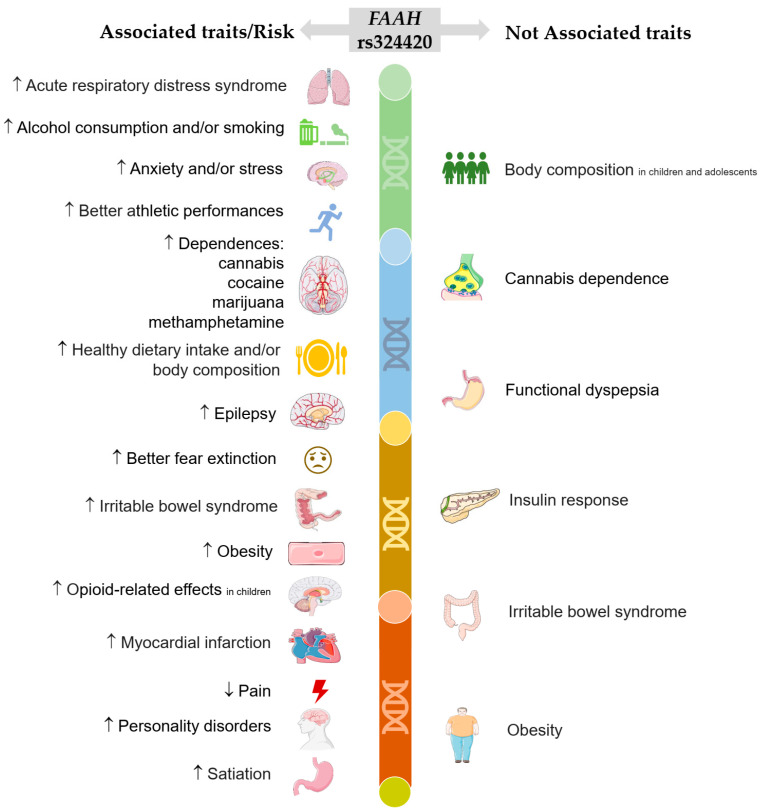
Scientific publications selected in PubMed reporting associated (*n* = 54) and not associated (*n* = 6) traits linked to psychobiological pathways of *fatty acid amide hydrolase* (*FAAH)* rs324420. Genetic variants of this polymorphism have been associated with the increased (↑) or decreased (↓) risk of experiencing various pathologies, disorders and/or behaviours, such as: (1) suffering acute respiratory distress syndrome [15]; (2) consuming alcohol and/or smoking [11,16,17,18,19,20,21]; (3) experiencing anxiety and/or stress [22,23,24,25]; (4) achieving better athletic performances [6,26,27,28]; (5) suffering substance dependences in adults, including cannabis [12,29,30,31,32,33], cocaine [34], marijuana [35,36,37,38] and methamphetamine use [39]; (6) practicing a healthier dietary intake and/or body composition [40,41,42,43]; (7) suffering neurological disorders, such as epilepsy [26]; (8) dealing better with fear extinction [44,45,46,47,48]; (9) suffering irritable bowel syndrome [13,49,50]; (10) being obese [51,52,53]; (11) experiencing opioid-related effects in children [54,55,56]; (12) suffering myocardial infarction [57]; (13) feeling less pain [58,59,60]; (14) having personality disorders [10,42]; and (15) feeling satiation more easily [61]. On the other hand, a few studies have not found associations between *FAAH* rs324420 variants and body composition in children and adolescents [62], cannabis dependence [29], functional dyspepsia [63], insulin response [64], irritable bowel syndrome [65] and obesity [66].

Given the potential biological impact of this SNP on pain and inflammation regulation, its roles in sports performance and sports medicine should be further dissected. Therefore, this narrative review aims to discuss: (1) the endocannabinoid system (ECS) and the biological pathways of *FAAH* rs324420, (2) the geographic distribution of this SNP, (3) its impact on elite sports performance, (4) its other psychobiological associations and (5) its implications for sports medicine. To do so, a search of the published literature that investigates the association between *FAAH* rs324420 and elite athletes was conducted by screening the PubMed platform. Data collection included scientific articles, books and book chapters published until 23 August 2023. The sole descriptor (i.e., keyword) that was used to search for the articles was “*FAAH* rs324420”. Both review and original articles were considered, totalling 60 publications. From these, findings of only 54 scientific publications were associated with the *FAAH* rs324420 at different levels. The associations between this polymorphism and different traits were investigated by diverse studies, which are summarised in Figure 1.

Supplemental publications were included by cross-referencing the reference lists of the retrieved articles. After data collection, the results were analysed through comprehensive reading and structured in accordance with the themes discussed in this article. A total of 131 publications involving human participants and written in one of five languages (Portuguese, English, Spanish, French or Italian) were included in this review.

## 2. The Endocannabinoid System and the Biological Pathways of *FAAH* rs324420

### 2.1. The Endocannabinoid System Signalling

**Figure 2:**
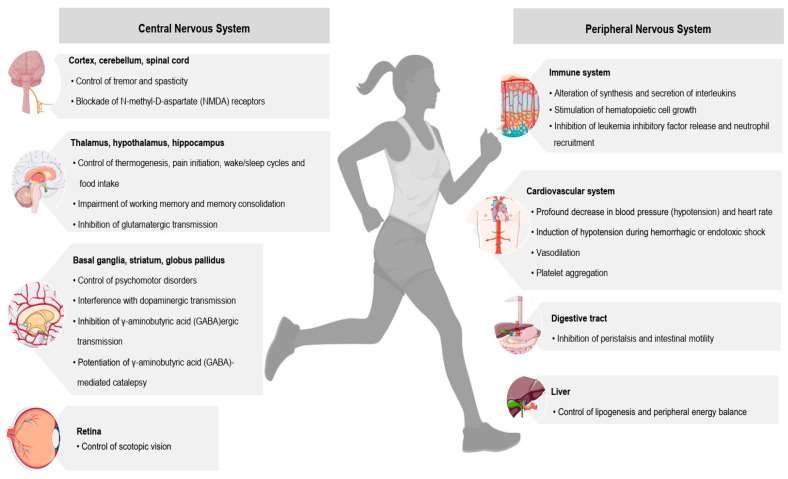
Effects of the endocannabinoid system in central nervous and peripheral systems (adapted from Wang, Dey and Maccarrone) [74]. The activation of several molecular targets by the endocannabinoid anandamide or the 2-arachidonoidglycerol results in a variety of biological actions, affecting practically all central and peripheral systems in animals, as shown.

One of the most promising biological systems involved in emotion control is the ECS. It is a highly complex signalling system mostly involved in body homeostasis due to its direct action on the central nervous system (CNS) [67]. Specifically, this system acts on immune response modulation [68], motor activity, fear and anxiety regulation [69,70], cardiorespiratory system control [71], stress responses [72], memory process [73] and pain perception [74] by the activation of several molecular targets by the AEA or the 2-AG, resulting in a variety of biological actions, as shown in Figure 2. Regarding its composition, ECS encompasses endocannabinoids (eCBs) that are neuromodulators of the CNS, the two most-studied being AEA and 2-arachidonoidglycerol (2-AG). These two act as endogenous ligands for two cannabinoid receptors, namely CB1 and CB2, and as proteins responsible for their biosynthesis, metabolism and release [75]. The eCBs can also function as immunomodulators, such as the AEA, which protects neurons from inflammatory damage during CNS inflammation [67].

The functioning of ECS depends on the interaction between the synthesis, release and inactivation of its endogenous agonists, the eCBs. Both AEA and 2-AG are synthesised via a phospholipid-dependent mechanism, released and taken up by cells via passive diffusion across the plasmatic membrane [67]. After their synthesis, they do not concentrate into synaptic vesicles and are primarily degraded by intracellular enzymatic hydrolysis by the *FAAH*, in the matter of AEA and by the monoacylglycerol lipase (MAGL) and *FAAH*, in the case of 2-AG [67]. In turn, AEA operates on CB1 receptors (R), which are largely found in the CNS, and CB2 receptors, which are predominantly found in the peripheral nervous system and involved in immunological response [66,76]. The CB1R is a G protein-coupled receptor integrated in presynaptic terminals on GABAergic and glutamatergic neurons and is thus integrated in the retrograde signalling of neurotransmission. It is significantly expressed in brain areas involved in emotional behaviour control and memory-related plasticity, namely the prefrontal cortex, amygdala and hippocampal formation [77].

### 2.2. FAAH Protein Functioning and FAAH Gene Variation

*FAAH* is a serine hydrolase that has been associated with the inactivation of the eCBs, which consists of transporting them back to the cell. This transport is contrary to the normal transport of other neurotransmitters, meaning without a difference in the sodium gradient (Na^+^). It is thought that it can occur using lipid transporter proteins by mechanisms of facilitated diffusion and in favour of the concentration gradient [78]. Once inside the cell, the AEA is hydrolysed by the enzyme *FAAH*.

This enzyme is encoded by the *FAAH* gene, which is positioned on the short arm (p) of chromosome 1 at position 33 (1p33). As mentioned, it is considered an intracellular enzyme that can hydrolyse AEA and other bioactive amides. It is also responsible for controlling the brain concentrations of these compounds, having a strategic location in the brain, although its presence has been verified in other tissues, for example, the liver, lungs, kidneys, spleen and testis. In the brain, namely in the hippocampus, cortex and cerebellum, this enzyme is located close to membranes of postsynaptic nerve terminals, where the CB1R are present. Therefore, its location indicates that *FAAH* plays an active role in AEA inactivation, which takes place in postsynaptic neurons [79].

### 2.3. FAAH rs324420 Polymorphism

There are sequence variations in different regions of *FAAH* [30,35,44,80,81,82,83], rs324420 being the most-studied SNP of this gene concerning different phenotypes and functions or disorders, such as obesity [84], mental diseases [85] or biopsychosocial disorder [86]. This polymorphism consists of the substitution of the nucleotide Cytosine (C) by an Adenine (A) at nucleotide position 385, translating into an amino acid exchange of a Proline (Pro) for a Threonine (Thr) in the codon 129, designated *FAAH* C385A (Pro129Thr; rs324420) (National Center for Biotechnology Information) [34]. This SNP has a minor allele frequency (MAF) of >5% [30]. The A allele, which is the minor, is known to reduce *FAAH* cellular activity and expression due to increased sensitivity to proteolytic degradation. Consequently, compared to C allele individuals, AA genotype carriers have almost 50% decreased *FAAH* activity, which translates into increased signalling and concentrations of AEA [23,87]. The A allele has been related to an increased risk for borderline personality disorder [10], substance use disorders [11,19], cannabis, methamphetamine, and cocaine dependence [27,28,33,34,38] and overweight/obesity [88,89]. However, contradictory results have been observed in cannabis users [12] and regarding athletes’ nutritional status [5,6].

## 3. Geographic Distribution of *FAAH* rs324420 Alleles

**Figure 3:**
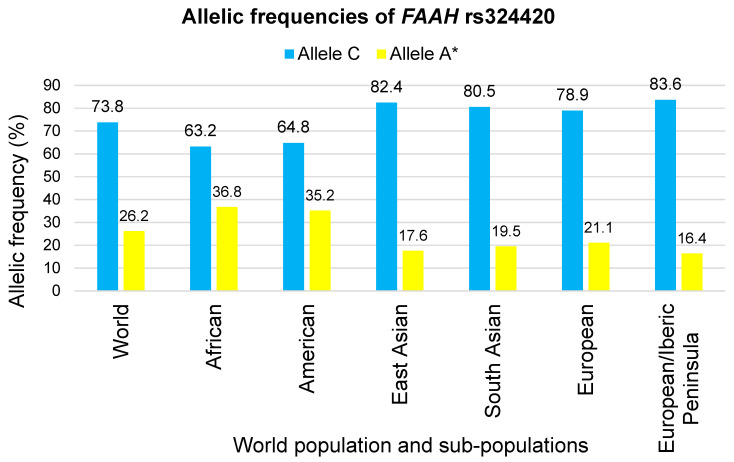
Geographic distribution of allelic frequencies of the *fatty acid amide hydrolase* (*FAAH)* rs324420 [90]. * Minor allele.

As mentioned, the rs324420 A allele has been associated with a lower expression of *FAAH* levels [89]. Its prevalence rates range from 36.8% and 35.2% in the African and the American populations, respectively, to 21.1%, 19.5% and 17.6% in the European, South and East Asian populations, respectively [90,91] (Figure 3). Given the recent findings involving Southwestern European athletes [5,6], it is important to note that the frequency of this allele in the Iberic peninsula is even lower (16.4%).

## 4. *FAAH* rs324420 Polymorphism and Elite Athletic Performance

The *FAAH* rs324420 polymorphism has been associated with pain tolerance and inflammation [92], both with strong implications for the athlete’s well-being and performance, especially among those competing at high-intensity and/or invasion/contact sports with a high frequency of injuries and/or traumas. However, the biological role of the C385A variant in sports performance is not yet a matter of consensus.

### 4.1. Biological Evidence in Elite Athletic Performance

First, evidence suggested a detrimental effect of the 385A allele (A allele), as it seemed to be more prevalent among sedentary people than in elite athletes [2,14]. A case-control study including 413 Polish Caucasian elite power and endurance athletes (aged 23.5 ± 4.7 years, of which 36.3% were females) and 451 Caucasian sedentary controls (aged 23.0 ± 3.1 years with 51.9% females) revealed that the polymorphism AA genotype was more common among controls than athletes and that elite sports performance was negatively affected by the AA genotype (AA versus (vs.) CC + AC; odds ratio (OR) = 0.44; 95% Cl, 0.24–0.81; *p* = 0.0084) [14]. Identical results were reported later in 621 elite athletes (183 of power, 212 of endurance and 226 combat sports) and 451 controls [2], where the AA genotype was underrepresented in both power (AA vs. CC + AC; OR = 0.36, 95% CI = 0.15–0.86, *p* = 0.017) and endurance athletes (AA vs. CC + AC; OR = 0.42, 95% CI = 0.20–0.90, *p* = 0.022) in comparison with controls. Furthermore, when the two groups of athletes were examined together, the effect on athletic status was even more pronounced (in the recessive model: OR = 0.40, 95% CI = 0.22–0.72, *p* = 0.002), indicating a negative impact on athletic performance [14].

Recently, a cross-sectional study conducted with 116 of the world’s best rink-hockey players (aged 28.2 ± 8.7 years), of which 15.5% were females (25.3 ± 7.9 years old) and 84.4% were males (28.8 ± 8.7 years old), found that the *FAAH* rs324420 and the presence of severe sports injuries were independent predictors of elite sports performance [5]. The A allele appeared to have a positive effect among elite players, as the carriers were three times as likely to be super athletes than those with the CC genotype (AA/AC vs. CC; adjusted OR = 2.88; 95% Cl, 1.06–7.80; *p* = 0.038), possibly due to better stress coping and higher pain tolerance. A later cohort study of 228 elite volleyball players (aged 26.7 ± 8.1 years old, 29.0%females) observed that carriers of the 385A allele were two times more likely to be super athletes than athletes demonstrating the CC genotype (adjusted OR = 2.00; 95% Cl, 1.04–3.82; *p* = 0.037) [6].

Given the inconsistent results regarding the C385A variant, validation studies considering other homogeneous sports modalities in addition to rink-hockey and volleyball, larger cohort samples and both male and female representation are crucial to investigate the specific role of this SNP in athletic performance. In addition, the athletes’ ethnicity may also influence the prevalence of *FAAH* rs324420 genotypes. Therefore, including homogeneous groups of athletes in this regard may enhance the research impact. For instance, the two first studies were conducted with athletes being exclusively of Polish Caucasian origin, whereas the two more recent studies mostly involved Portuguese people, with almost one-third being of other nationalities (Spanish, Argentinian, Italian, French, Servian, American, Canadian, Brazilian, Chilean, Mozambican, Angolan and Australian) (Table 1).

### 4.2. Biological Pathways, Elite Athletic Performance and Sport Medicine

In addition to their demands regarding training routines and the need for high achievements in international and national competitions, athletes also face very stressful environments daily [89]. Their stress response can be influenced by the mechanism of action of *FAAH* through the ECS, which is corroborated by the recently observed impact of *FAAH* rs324420 on elite athletic performance [93,94].

Stress is described as the body’s reaction to an internal or external stimulus to prepare for potential injuries and/or diseases. Physical and psychological stress causes a series of responses that produce immediate threat management followed by a return to homeostasis. The first brain responses are released in a few seconds of the stimulus [89]. This mechanism involves several neurotransmitters, including serotonin, noradrenaline, the fast-acting stress hormone adrenaline, GABA and glutamate. Endocrine reactions begin minutes to hours after the stressful stimulus, driven by stimulation of the HPA axis and culminating in the production of adrenal glucocorticoids. Preclinical results clearly suggest the concept that stress alters eCB signalling and that this is a fundamental mechanism through which stress alters synaptic plasticity in diverse brain areas [89].

**Figure 4:**
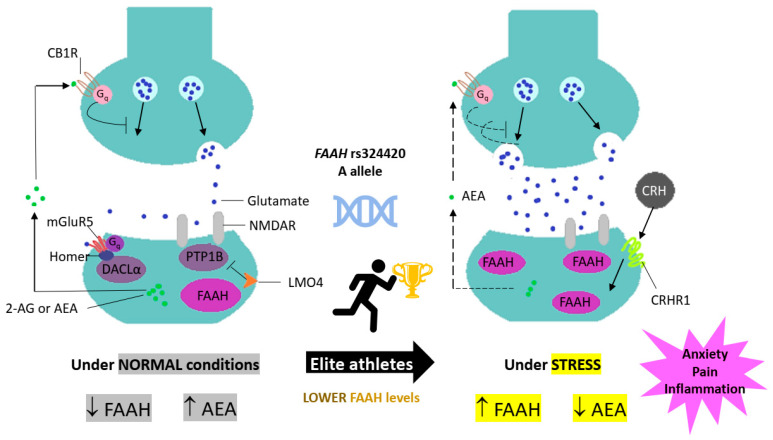
The endocannabinoid system plays a key role in controlling how highly skilled athletes react to stress. Under normal circumstances, the endocannabinoid system modulates synaptic function by inhibiting the release of the neurotransmitter glutamate via *N*-arachidonoylethanolamine (AEA). However, under severe stress, the corticotropin-releasing hormone (CRH) and its receptor (corticotropin-releasing hormone receptor 1-CRHR1) can be activated, increasing (↑) the basolateral amygdala’s fatty acid amide hydrolase (*FAAH*) activity. As a result, AEA levels drop (↓) and lose their ability to control glutamate release. Consequently, enhanced anxiety-like behaviour is caused by increased neuronal excitability in the basolateral amygdala (adapted by Silva et al.) [6]. CB1R: cannabinoid type 1 receptor; Gq: family G protein; mGluR5: metabotropic glutamate receptor 5; 2-AG: 2-arachidonoyl glycerol; DAGLα: diacylglycerol lipase-α; PTP1B: protein tyrosine phosphatase 1B; NMDAR: NMDA receptor; LMO4: LIM domain only 4.

Under stress conditions (Figure 4, adapted from Silva et al.) [6], *FAAH* is triggered to breakdown the AEA, raising neuronal excitability in the amygdala, a critical anxiety-mediating part of the brain [2]. In contrast, inhibiting *FAAH* reduces anxiety-like behaviour [89] and may provide an antidepressant effect by stimulating the CB1 receptor [95]. As a result of a higher susceptibility to *FAAH* degradation [2], the SNP A allele may be associated with faster habituation of amygdala responsiveness to danger/threat, lower anxiety-like behaviour and greater fear-extinction learning. This is critical for elite athletes, who need to present personality attributes related to stress response and effective mental discipline to deal with uncertain events more rapidly, boost their motivation for sports competition and decrease the risk of sports injuries [14,78,89].

## 5. *FAAH* rs324420 Polymorphism and Other Psychobiological Associations

### 5.1. FAAH rs324420 Polymorphism, Stress, Anxiety and Fear Extinction

The exposure to repeated stressful situations results in the habituation of the HPA axis activation and the behavioural stress response. The ability to habituate to repeated exposure to a non-threatening stimulus is protective because it avoids the repercussions of prolonged stress [5,6]. The potential of eCB-mediated synaptic plasticity to facilitate habituation may be one of the most important roles of this mechanism in the setting of human psychopathology [89].

Stress and glucocorticoids both raise 2-AG levels in the hypothalamus, hippocampus, prefrontal cortex and raphe nuclei. When a plasma membrane-associated glucocorticoid receptor in the hypothalamus is activated, 2-AG levels rise rapidly, inhibiting glutamate release [96]. The mechanism by which glucocorticoids increase 2-AG levels in the prefrontal cortex is still unknown, although this increase is known to inhibit GABA release [97]. Activation of CB1R signalling is essential for glucocorticoid-mediated feedback inhibition of the HPA axis [98]. Therefore, the eCB system enhances the activation of resilience elements during and/or after stress exposure [99].

As mentioned before, acute stress modifies the concentrations of the two primary eCBs, AEA and 2-AG, in the brain, altering CB1R signalling [5,6,89]. Acute stress reduces AEA concentrations in the amygdala and prefrontal cortex; these changes are accompanied by an increase in *FAAH* activity and are mediated by CRH effects that modify *FAAH* activity [100]. Reduced AEA concentrations in the amygdala allow activation of the HPA axis, while *FAAH* inhibition lowers the glucocorticoid response [101]. A study conducted with 661 total participants (19.6 ± 1.2 years old), 121 of whom had at least one Diagnostic and Statistical Manual of Mental Disorders-IV diagnosis, concluded that individuals with high AEA inhibitory tone (*FAAH* 385A allele carriers) and high corticotropin-releasing hormone (CRH) signalling (corticotropin-releasing hormone receptor type 1 (CRHR1) rs110402 A allele homozygotes) had the least temporal habituation of the basolateral amygdala, a neuroimaging associated with fear extinction [24]. Stress-induced CRH signalling via CRHR1 in the basolateral amygdala increases *FAAH* activity [102]. In turn, increased activity of this catabolic enzyme leads to the reduction of AEA and a deprivation of inhibitory tone, which is required for lowering anxiety and sustaining fear extinction [78].

When compared to CC homozygotes, healthy individuals carrying the *FAAH* rs324420 A allele demonstrated enhanced fear extinction learning [44] and lower anxiety levels [78,103,104,105]. This impact was more pronounced in AA homozygotes, who had a simpler degradable *FAAH* enzyme, resulting in higher AEA among carriers [106]. In a sample of 55 healthy male adults, including 17 AC genotype carriers and 34 CC homozygotes, brain activation upon an unextinguished versus extinguished stimulus was greater in AC genotype carriers than in CC homozygotes in core neural elements related to extinction recall. They also displayed higher AEA levels and lower anxiety levels (*p* < 0.05) [22]. However, controversial results have been found. In a study with 928 Hungarian (all Caucasians) subjects (31.3 ± 10.5 years old; 69.8% females), *FAAH* C385A A allele carriers who experienced childhood adversities demonstrated higher levels of anxiety than CC carriers (*p* = 0.0023) [23]. These findings may be due to a decreased CB1R receptor expression during neurodevelopment in the human brain caused by childhood traumas influencing affective phenotypes, namely the *FAAH* C385A polymorphism [23].

### 5.2. FAAH rs324420 Polymorphism, Pain and Inflammation

In response to stress and injuries, ECS has been studied as a key target related to endogenous analgesia [107]. However, very few studies have explained the relationship of *FAAH* rs324420 with pain [60]. The *FAAH* influences eCB concentrations in peripheral and central neurological systems, including immunological cells. It is involved in nociception, inflammatory reactions and a variety of other processes [108,109,110,111]. Inhibiting *FAAH*’s enzymatic activity extends the action of AEA and hence improves eCB-mediated antinociception [60,107].

The amino acid mutation P129T (SNP rs324420) lowers *FAAH* protein expression via a posttranslational system that has not been sufficiently explained. As a result, the SNP rs324420 is the most likely candidate to be the causative variable underlying the connection with sensitivity to cold pain [107]. In a study with women aged 18 to 75 years (900 were tested for cold pain and 1000 for sensitivity to heat pain) and who underwent surgery for breast cancer, patients were divided by the *FAAH* rs324420 genotype (72 for A/A, 380 for A/C and 471 for C/C) [108]. A significant association between the SNP and cold pain sensitivity was found, with greater association in subjects homozygous for the minor allele (AA genotype), who reported less sensitivity to cold pain (β = −1.48; 95% CI −2.14 to −0.8) than other groups [60].

Given the association of the A allele of the *FAAH* rs324420 with lower *FAAH* activity, 21 highs (with significantly greater pain reduction than lows), 66 low hypnotizable subjects (lows) and 172 controls were genotyped [58]. The A allele frequency increased from lows to controls and from controls to highs (best fitting curve: logarithmic model, F = 621.93, R2 = 0.998, *p* = 0.026). Therefore, the role of the *FAAH* polymorphism in high analgesia should not be ruled out, as eCB minor variations can be magnified by eCB interactions with other neurotransmitters [58], as also demonstrated by other researchers [59].

The baseline amount of AEA release in the brain is modest, and neuronal secretion requires a trigger [79]. A powerful stimulus activates the stress response, which can be mediated by the ECS and endogenous opioids and induce stress-analgesia [112]. It seems that the AEA combined with an *FAAH* inhibitor can generate considerable antinociception. As a result, subjects having a mutation that is expected to reduce *FAAH* function and so prolong the AEA effect reported lower pain intensities and tolerance to cold pain [60].

### 5.3. FAAH rs324420 Polymorphism and Neural Dysfunctions

The *FAAH* rs324420 polymorphism has also been related to other neural dysfunctions, such as epilepsy and attention deficit hyperactivity disorder (ADHD). It has been suggested that an altered eCB system can have a neuroprotective effect by activating CB1 receptors by eCBs and selective CB1 agonists, but blocked CB1 receptors by specific antagonists may improve epileptogenesis and lead to diverse neurological conditions such as epilepsy [113] and ADHD [114].

Epilepsy is one of the most prevalent neurological disorders (between 7.60 per 1000) [115] that can be manifested as idiopathic generalised epilepsy and focal epilepsy [116], and it is mainly produced by gene changes and environmental influence. ADHD has a 2–7% global prevalence [117], with candidate gene pathways being influenced by some types of medication administrated for ADHD treatment [118].

As mentioned before, the *FAAH* C385A is responsible for an enlarged vulnerability of the *FAAH* enzyme to proteolytic degradation [76], which increases eCB, preventing the neurotoxicity caused by seizures [119]. Although the literature has investigated the protective effect of *FAAH* inhibitors in the brain area, a recent study conducted with a group of 250 epilepsy individuals, 157 cases with ADHD and 386 healthy controls [26] demonstrated that reduced levels of *FAAH* enzyme produced by this polymorphism increased generalised epilepsy risk by approximately two times (*FAAH* C384A genotype, OR = 1.755, 95% CI 1.124–2.742, *p* = 0.013, and allele, OR 1.462, 95% CI 1.006–2.124, *p* = 0.046). This may be due to potential differences in ligand/receptor ratios of the eCB system [120], as generalised epilepsy affects the broad brain region. In contrast, this SNP was not linked with the risk of ADHD.

The *FAAH* rs324420 variant has also been linked to substance use disorders, specifically cannabis dependence, and that altered *FAAH* activity has been shown to influence alcohol use [20,27], although findings are still complex and controversial [18]. A case-control study with 531 Greek participants (251 alcohol-dependent cases, mean age of 43.5 ± 11.5 yrs., 60 females and 91 males, and 280 controls, mean age of 42.8 ± 14.3 years, 92 females and 188 males) investigated the SNP in patients with Alcohol Use Disorder (AUD) [18]. The authors observed that the A allele was associated with an increased risk of AUD (OR = 0.55, 95% CI 0.41–0.73, *p* < 0.0001). This could serve as a potential biomarker for AUD susceptibility. Another study observed an increased risk of the slow *FAAH* activity group (C/A or A/A) with binge drinking (OR = 2.16, 95% CI 1.36–3.42 at 20 yrs. old, and OR = 1.61, 95% CI 1.10–2.36 at 30 yrs. old), drinking initiation (OR = 1.39, 95% CI 1.09–1.77) and escalation (OR = 2.24, 95% CI 1.05–4.76) and cigarette smoking initiation (OR = 1.20, 95% CI 1.04–1.39), but not with early smoking milestones [17]. A Greek study with 531 participants (251 alcohol-dependent subjects and 280 healthy participants) observed an increased risk of AUD among those carrying the SNP A allele (OR = 0.55, CI 0.41–0.73, *p* < 0.0001) [18]. Regarding alcohol dependence, Sloan et al. [20] observed that, in comparison to people with the CC genotype, American European adults with the A allele exhibited a higher frequency of compulsive drinking behaviours. Also, adolescents carrying AC and AA genotypes showed abnormal drinking attitudes and increased AUD scores [11]. However, due to the complex aetiology of AUD and diversity of genetic and environmental factors [18], further investigation with larger sample sizes and diverse populations are required to examine these findings.

A recent systematic review found that *FAAH* protein contributes to biological and clinical aspects of AUD and that pharmaceutical targeting of this molecule could be useful for alcohol withdrawal by reducing anxiety and resumption of alcohol intake [121]. Since *FAAH* affects brain reward signalling by metabolizing AEA, it might potentially increase addiction vulnerability [122]. As a result, the SNP rs324420 decreases *FAAH* catalytic activity and alters the addictive properties of a variety of substances [20]. The relationship between the SNP and substance use disorders [123] is consistent with previous research that found genetic links between methamphetamine [39], marijuana [36], cannabis [27] and cocaine [34].

## 6. Implications for Sport Medicine

The *FAAH* rs324420 may play diverse functions depending on athletes’ age, sex, ethnicity, performance level and type of sport (e.g., athletes submitted to high mechanical impacts may be at greater risk of sport-related injury and medical complications) [5]. Specific attention should be given to the A allele as it was associated with unique athletic achievements [5,6].

Given that the *FAAH* is a good candidate gene for drug discovery in patients dealing with inflammation and pain [124], medical staff working with sports injury prevention and recovery, or athletes’ illnesses should advise coaches regarding identification and selection of preventative strategies to be applied in training activities through an individualised training programme [125]. Furthermore, ethical procedures must be respected and followed as the imposition of genetic testing is potentially abusive [125].

While significant sex differences in rs324420 genotype frequencies have not been found in elite athletes [5,6], it seems that oestrogens may modify emotional behaviour by dysregulating the *FAAH* enzyme, increasing the ECS signalling and, as a result, decreasing women’s anxiety [95]. Thus, additional research with female athletes is required to corroborate this hypothesis.

Although scientific studies that genotyped elite athletes for the *FAAH* rs324420 have mostly involved Caucasian players [2,5,6,14], current world records differ by sex and ethnicity, with African ancestry athletes posting faster times in the 100 m, 200 m and 400 m than their Caucasian counterparts [126]. Given the evidence that players carrying the A allele (AA or AC genotype) are two or three times more likely to be super athletes than subjects with the CC genotype [5,6], this might be important for multidisciplinary teams (coaches and clinical staff) responsible for planning athletes’ training sessions and preparing them for and helping them recover from competitions, respectively.

The negative impact of sports injuries on athletes’ health and performance is undoubtable and can be very devasting, especially if they are recurrent. Therefore, mental qualities are essential to cope with injuries [127,128,129,130], acquire pain tolerance [4,131] and be resilient [2,5,6]. The limited scientific literature has shown that the *FAAH* rs324420 input, which codes for physiological aspects of brain regions related to psychobiological qualities, can be a helpful tool for athletic performance [6]. In fact, severe sports injuries and *FAAH* rs324420 were independent predictors of elite athletic performance, probably due to significant differences between athletes’ sex and training, as recently reported [5].

Sport medicine specialists and technical staff should be aware that athletes with genetically reduced *FAAH* activity and who are repeatedly submitted to stress (permanently elevated AEA) during their childhood may be vulnerable to anxiety and depression in later life due to long-term effects on stress response possibly by the CB1R downregulation throughout brain neurodevelopment [23].

## 7. Conclusions and Future Directions

The observed effect of the *FAAH* rs324420 is of the utmost importance for future research in elite athletes experiencing daily stressful training and competition events and potential sports injuries due to the mechanism of action of *FAAH* through the ECS applied to stress management, pain regulation and inflammation control. In addition to the promising character of *FAAH* for drug discovery in patients affected by inflammation and pain, its rare genetic variants may also improve mental discipline and physical performance among elite athletes. The review of the current literature presented in this article suggests that the role of genes coding for structural and biochemical components of brain areas related to psychological traits have been less investigated than athletes’ phenotypes related to musculoskeletal and cardiovascular functions. Nevertheless, genetic research in the sports context has shown modest progress since gene-based association analyses need to be more robust in discovering several minor and cumulative gene effects. In the future, more effective genomic-based research methodologies might speed up the discovery of genes associated with both mental and physical athletic performance and welfare. In addition, it will be critical to create cohorts of truly elite individuals with adequate specific physiological data to offer the requisite resolution, to investigate the findings using multidisciplinary methodologies and to enhance biological and clinical research on athletic ability, health and potential risk of disease or injury. This may impact teams’ (e.g., athletes, coaches and sports medicine staff) daily work, which can benefit from individualised training programmes according to each athlete’s sex, body composition, nutrition, previous injuries and environmental conditions, while helping to avoid eventual burnout and potential sport dropout.

## Figures and Tables

**Table 1:** Distribution of *FAAH* rs324420 genotypes among research studies involving elite athletes.

Authors	Population	Athletes’ Profile	Genotypes, *n* (%)		
			AA	AC	CC
Silva et al. [6]	75.1% Portuguese and 24.9% Others	Elite volleyball players (*n* = 219)	11 (5.0)	74 (33.8)	134 (61.2)
	75.8% Portuguese and 24.2% Others	Female (*n* = 66)	3 (5.0)	20 (33.3)	37 (61.7)
	74.8% Portuguese and 25.2% Others	Male (*n* = 162)	8 (5.0)	54 (34.0)	97 (61.0)
Silva et al. [5]	82.8% Portuguese and 17.2% Others	Elite rink-hockey players (*n* = 116)	4 (3.4)	34 (29.3)	78 (67.2)
	All Portuguese	Female (*n* = 18)	1 (5.6)	6 (33.3)	11 (61.1)
	79.6% Portuguese and 20.4% Others	Male (*n* = 98)	3 (3.1)	28 (28.6)	67 (68.4)
Peplonska et al. [2]	All Caucasians of Polish origin	Elite athletes (*n* = 621, 29.3% females)	27 (4.3)	259 (41.7)	335 (54.0)
		Power (*n* = 183)	6 (3.3)	79 (43.2)	98 (53.5)
		Endurance (*n* = 212)	8 (3.8)	84 (39.6)	120 (56.6)
		Combat (*n* = 226)	13 (5.8)	96 (42.4)	117 (51.8)
Peplonska et al. [14]	All Caucasians of Polish origin	Elite athletes (*n* = 413, 36.3% females)	16 (3.9)	169 (40.9)	228 (55.2)
		Power (*n* = 188)	6 (3.2)	80 (42.6)	102 (54.2)
		Endurance (*n* = 225)	10 (4.4)	89 (39.6)	126 (56.0)

## Data Availability

All data have been included in the manuscript.
